# Universal compilation for quantum state tomography

**DOI:** 10.1038/s41598-023-30983-4

**Published:** 2023-03-06

**Authors:** Vu Tuan Hai, Le Bin Ho

**Affiliations:** 1 University of Information Technology, Ho Chi Minh City, 700000 Vietnam; 2 Vietnam National University, Ho Chi Minh City, 700000 Vietnam; 3 Ho Chi Minh City Institute of Physics, National Institute of Applied Mechanics and Informatics, Vietnam Academy of Science and Technology, Ho Chi Minh City, 700000 Vietnam; 4 Frontier Research Institute for Interdisciplinary Sciences, Tohoku University, Sendai, 980-8578 Japan; 5 Department of Applied Physics, Graduate School of Engineering, Tohoku University, Sendai, 980-8579 Japan

**Keywords:** Quantum mechanics, Quantum metrology, Qubits

## Abstract

Universal compilation is a training process that compiles a trainable unitary into a target unitary. It has vast potential applications from depth-circuit compressing to device benchmarking and quantum error mitigation. Here we propose a universal compilation algorithm for quantum state tomography in low-depth quantum circuits. We apply the Fubini-Study distance as a trainable cost function and employ various gradient-based optimizations. We evaluate the performance of various trainable unitary topologies and the trainability of different optimizers for getting high efficiency and reveal the crucial role of the circuit depth in robust fidelity. The results are comparable with the shadow tomography method, a similar fashion in the field. Our work expresses the adequate capability of the universal compilation algorithm to maximize the efficiency in the quantum state tomography. Further, it promises applications in quantum metrology and sensing and is applicable in the near-term quantum computers for various quantum computing tasks.

## Introduction

Quantum computers promise an excellent computational capacity that is intractable for classical computers to solve challenging problems, including materials science^1–3^, information science^4,5^, computer science^6,7^, mathematical science^8–10^, and others. However, there are two major challenges to bringing quantum computers to materialize^2^: (1) it is difficult to access full information from entangled systems because of the state collapse upon measurements, and (2) it is difficult to build, control, and measure quantum states with arbitrarily high accuracy. In this regard, even though the current state-of-the-art quantum computers rely on the noisy intermediate-scale (NISQ devices,) which usually prevent high efficiency^11^, various hybrid quantum-classical algorithms were proposed and actively studied recently^12^, and that could be promising for quantum speedup in the regime of NISQ devices. Massive applications including variational quantum eigensolvers^13–17^, quantum approximate optimization algorithms^18^, new frontiers in quantum foundations^19–22^, and others, were reported.

Beyond the actively studied VQAs, the universal compilation has drawn tremendous interest recently. Its core idea relies on a training process to transform a trainable unitary into a target unitary^23,24^. It was demonstrated in different applications from gate optimization^23^, to quantum-assisted compiling process^24^, continuous-variable quantum learning^25^, and robust quantum compilation^26^. The future of universal quantum compiling could be circuits depth-compression, black-box compiling, error mitigation, gate-fidelity benchmarking, and efficient gate synthesis.

In another aspect, quantum state tomography (QST) is a measurement process performed on numerous identical copies of a system to extract its state’s information^27^. In general, for a given unknown quantum state $|\psi \rangle$ in a complex Hilbert space of *d*-dimension, it requires an exponentially growing $2^d-1$ measurements on different bases to completely reproduce the state, which is intractable for large systems. Numerous methods were proposed for improving the standard QST in terms of efficiency^28–31^, methodology^32–38^, quantum dynamic^39–41^, and so on. Recently, the quantum circuits-based QST has attracted significant attention owing to the incredible advantages of the quantum device^42–44^, which allows to efficiently prepare quantum states with high confidence, fully control the Hamiltonian for the state evolution, and directly access the measurement results. A variational approach^45^ and single-shot measurements^46,47^ to name a few, were investigated.

Despite recent achievements on the QST, it is still challenging to implement in the NISQ devices. In this work, we introduce a promising application of the universal compilation on the QST. Our main idea is to use a trainable unitary acting upon a known fiducial state to reconstruct an unknown state, which is created by using a Haar random target unitary acting upon the fiducial state. The advantage of this method is that it requires low-depth trainable unitaries and few measurements to realize the target state, which significantly reduces the complexity and allows for tractability of large systems. Furthermore, the flexibility of the trainable unitaries is more elevated than that of the target unitaries, resulting in a better fault-tolerant capacity and thus allowing high efficiency for the trainable quantum circuits.

**Figure 1:**
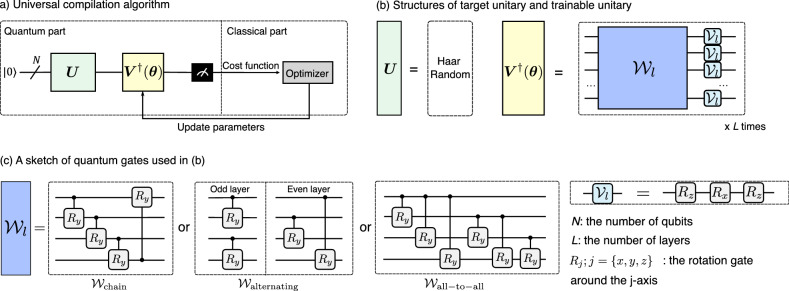
Universal compilation-based quantum state tomography. (**a**) A universal compilation algorithm consists of a quantum part and a classical part. In the quantum part, a final state is created by applying a set of quantum gates $\varvec{U}$ followed by $\varvec{V}^\dagger$ onto the initial circuit and then be measured. In the classical part, we compute the appropriate cost function, use an optimizer to compute new parameters, and update the scheme until it converges. (**b**) Structures of the target and trainable unitaries used in the QST. The unitary $\varvec{U}$ is a Haar random generator while $\varvec{V}^\dagger$ is parameterized into $\varvec{V}^\dagger (\varvec{\theta })$ and broken out into entangled gates $\mathcal {W}$ and local rotation gates $\mathcal {V}$ with several structures as shown in the figure. (**c**) A sketch of some quantum gates used in (**b**). Other notations: *N* the number of qubits, *L* the number of layers, $R_j, j = \{x, y, z\}$ the rotation gate around the *j* axis.

Concretely, we first introduce the general framework of the universal compilation-based quantum state tomography (UC-QST) We also introduce several gradient-based optimizers, including the standard gradient descent (SGD), the Adam, and the quantum natural gradient descent (QNG). We discuss the numerical experiment results for a representative case of single-qubit tomography, and then evaluate the reconstructing efficiency of unknown Haar random states via various popular circuit ansatzes. We find that the circuit depth plays a crucial role in the robust fidelity, i.e., by choosing a proper circuit depth via the number of layers in the quantum circuit, we get high fidelity at any qubit numbers. We finally compare the results with the shadow tomography method^48,49^, a similar fashion in the field.

The study reveals that the accuracy mainly relies on (1) the ansatz topologies with the optimal circuit depth and (2) the significant impact of different optimizers. Our study can further promise applications in quantum metrology and sensing, and new frontier foundation aspects. Moreover, it is possible to implement the algorithm on near-term quantum computers, and thus it could be a valuable technique for verifying the fidelity of quantum circuits and studying various quantum computing tasks. These are also benefits that overcome the standard QST, which requires the set-up of traditional experiments, consumes heavy post-processing calculations to reproduce the quantum state, and the accuracy depends on the estimators, such as the Maximum-Likelihood and Least-Squares^27^.

## Results

We introduce a universal compilation scheme^23–26^ to translate a given state into another one and apply it to quantum state tomography.

### Universal compilation-based quantum state tomography (UC-QST)

A universal compilation scheme consists of a quantum part and a classical part, as shown in Fig. 1a. The quantum part is a circuit with parameterizable ansatzes. Let $\varvec{U}$ is a fixed target unitary and $\varvec{V}^\dagger (\varvec{\theta })$ is a trainable unitary ansatz (sets of quantum gates with some parameters $\varvec{\theta }$) that act sequentially onto the circuit and transform an initial state $|\psi _0\rangle$ into a final state $|\psi _f\rangle$ as

1 $$\begin{aligned} |\psi _{f}\rangle = \varvec{V}^\dagger (\varvec{\theta }) \varvec{U}|\psi _0\rangle . \end{aligned}$$

The transition probability yields

2 $$\begin{aligned} p(\psi _0\rightarrow \psi _f) = \big |\langle \psi _0|\psi _f\rangle \big |^2 = \big |\langle \psi _0|\varvec{V}^\dagger (\varvec{\theta }) \varvec{U}|\psi _0\rangle \big |^2. \end{aligned}$$

Our task is to maximize the transition probability $p_{\mathrm{max}}(\psi _0\rightarrow \psi _f)$, such that a state $|\psi \rangle \equiv \varvec{U}|\psi _0\rangle$ is compiled to $|\phi (\varvec{\theta })\rangle \equiv \varvec{V}(\varvec{\theta })|\psi _0\rangle$. The maximization of the transition probability, i.e., $p(\psi _0\rightarrow \psi _f) = 1$, implies $|\psi \rangle = |\phi (\varvec{\theta })\rangle$, which can be applied to the QST as we will describe below.

Concretely for the QST, let $|\psi _0\rangle \equiv |\varvec{0}\rangle = |0\rangle ^{\otimes N}$, where *N* is the number of qubits, we transform it into a random (unknown) quantum state $|\psi \rangle = \varvec{U}|\varvec{0}\rangle ,$ via a Haar random unitary $\varvec{U}$^50^. To reconstruct this state, we apply a trainable unitary evolution $\varvec{V}^\dagger (\varvec{\theta })$ that can learn the role of $\varvec{U}$, i.e., a reconstructed state $|\phi (\varvec{\theta })\rangle = \varvec{V}(\varvec{\theta })|\varvec{0}\rangle$ resembles to the unknown state $|\psi \rangle$, where $\varvec{\theta }= \{\theta _1, \theta _2,\ldots , \theta _M\}$ can be adaptively updated during a training process, *M* is the number of trainable parameters. There is no free lunch for the choice of $\varvec{V}^\dagger (\varvec{\theta })$^25^. However, it can break out into a sequence of single-qubit and multi-qubit gates as

3 $$\begin{aligned} \varvec{V}^\dagger (\varvec{\theta }) = \prod _{l = 1}^L \mathcal {V}_l(\varvec{\theta }_l) \mathcal {W}_l(\varvec{\theta }_l); \text { with } \mathcal {V}_l = R_zR_xR_z, \end{aligned}$$

as shown in Fig. 1b, wherein $\mathcal {W}_l$ includes the chain, alternating, and all-to-all structures^51^ as shown in Fig. 1c. We emphasize that the entangled gates $\mathcal {W}$ consist of two-qubit controlled *y*-rotation gates, which differs from previous works^51^. We refer to these gates as parameter-dependent entanglement gate. They are useful for preparing variational states metrology^21^ and rapid entangled circuits^52^, for testing of the expressibility and entangling capability^53^, and so on.

To qualify how closed the two states are, we consider the Fubini-Study distance as^54^

4 $$\begin{aligned} d(\psi ,\phi (\varvec{\theta })) = \sqrt{1-|\langle \phi (\varvec{\theta })|\psi \rangle |^2} = \sqrt{1-p_0(\varvec{\theta })}, \end{aligned}$$

where $p_0(\varvec{\theta }) = |\langle \phi (\varvec{\theta })|\psi \rangle |^2 = |\langle \varvec{0}|\varvec{V}^\dagger (\varvec{\theta }) \varvec{U}|\varvec{0}\rangle |^2$ is the probability for getting the outcome $|\varvec{0}\rangle$. In the quantum circuit, we apply a sequence of $\varvec{U}$ followed by $\varvec{V}^\dagger (\varvec{\theta })$ onto the initial state $|\varvec{0}\rangle$ to get the final state $\varvec{V}^\dagger (\varvec{\theta }) \varvec{U} |\varvec{0}\rangle$ and then measure a projective operator $\varvec{P}_0 = |\varvec{0}\rangle \langle \varvec{0}|$, which yields the probability $p_0(\varvec{\theta })$.

The variational (reconstructed) state becomes the target (unknown) state if the distance reaches zero. In the classical part, we thus use the Fubini-Study distance as a cost function that needs to minimize, i.e., $\mathcal {C(\varvec{\theta }}) = d(\psi ,\phi (\varvec{\theta }))$, such that

5 $$\begin{aligned} \varvec{\theta }^*= \mathop{argmin}\limits_{\varvec{\theta }}^{} \mathcal {C}(\varvec{\theta }). \end{aligned}$$

By training the variational circuit until it converges, we obtain the optimal $\varvec{\theta }^*$ and the reconstructed state yields $|\phi (\varvec{\theta }^*)\rangle = \varvec{V}(\varvec{\theta }^*)|\varvec{0}\rangle$. This is a normalized pure state because $\varvec{V}(\varvec{\theta }^*)$ is a unitary ansatz, i.e., $\varvec{V}^\dagger (\varvec{\theta }^*)\varvec{V}(\varvec{\theta }^*)=\varvec{I}$.

For the training process, we apply gradient-based optimizations to iteratively update the parameters $\varvec{\theta }$ and minimize the cost function. We first compute the derivative $\partial _{\theta _j}\mathcal {C}(\varvec{\theta })$ for all $\theta _j\in \varvec{\theta }$ and then compute new parameters via various appropriate optimizers, including the Standard gradient descent (SGD), Adam gradient descent^55^, and Quantum natural gradient (QNG)^56^. See “Methods” section for details.

**Figure 2:**
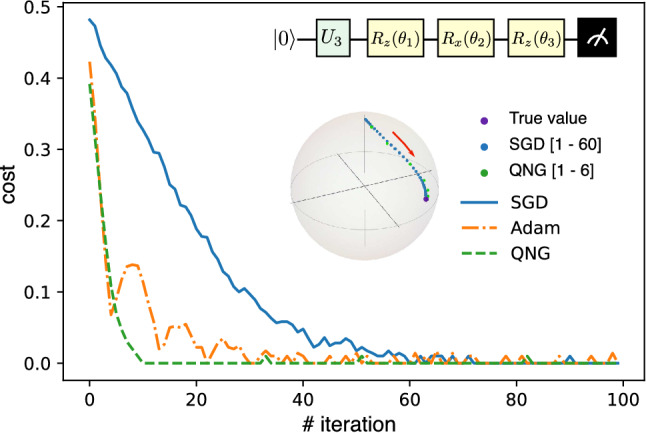
Single-qubit tomography. Cost function versus the number of iterations for difference optimizers: SGD (blue), Adam (orange), and QNG (green). Inset 1: quantum circuit for a single-qubit tomography where an unknown qubit state is generated by a random unitary $\varvec{U}_3\left( \theta ,\phi ,\lambda \right)$, and $V^\dagger (\varvec{\theta })$ is made up of $R_z(\theta _{1})$, $R_x(\theta _{2})$, and $R _z(\theta _{3})$ gates. Inset 2: Bloch sphere represents the qubit states: violet circle: the true state, blue circle: the trajectory of the reconstructed state under the SGD optimizer for the iterations run from 1 to 60, green circle: the trajectory under the QNG optimizer for the iteration runs from 1 to 6.

**Figure 3:**
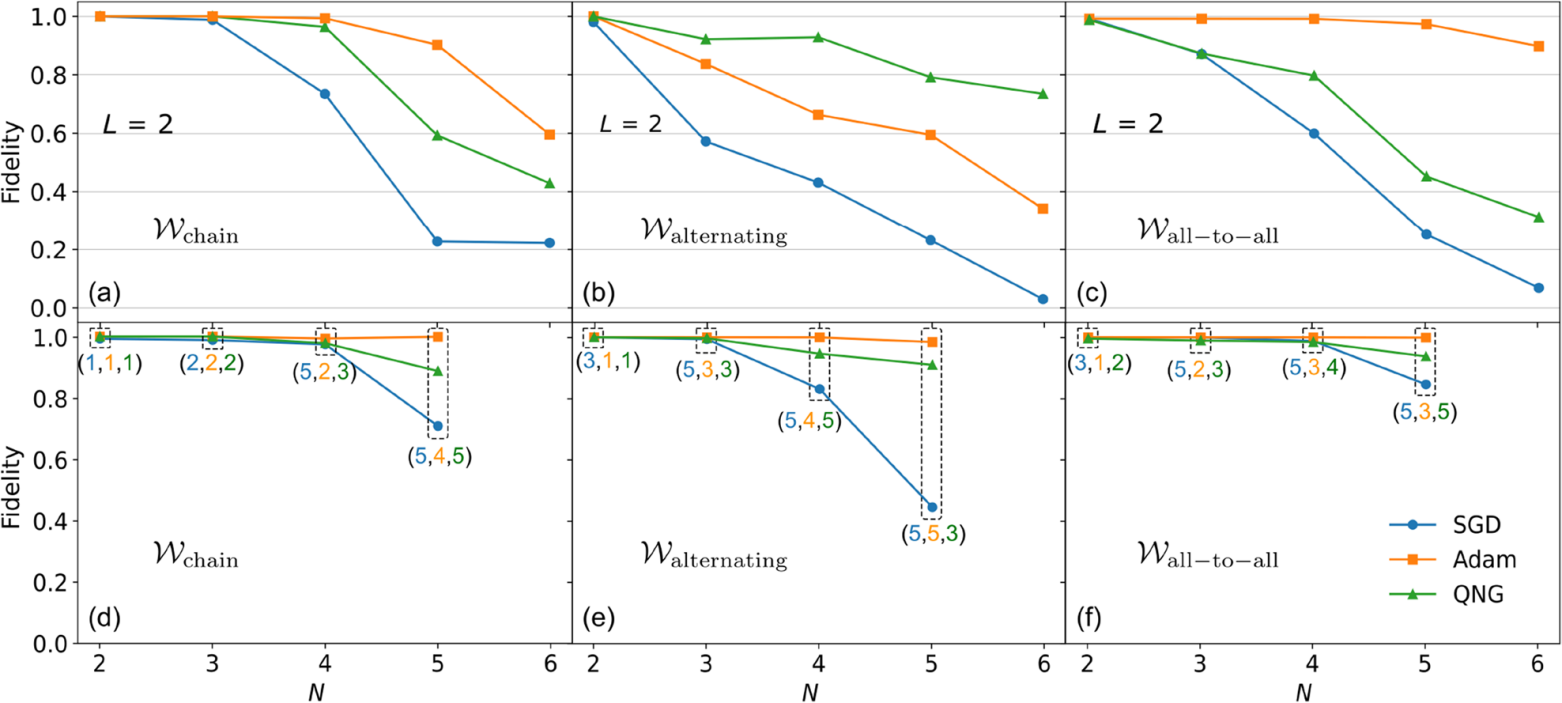
Numerical results for quantum state tomography. (**a**–**c**) Plot of the fidelity between an unknown Haar random state $|\psi \rangle$ and its reconstructed state $|\phi (\varvec{\theta })\rangle$ for different $\mathcal {W}$ structures: $\mathcal {W}_{\mathrm{chain}}$ (**a**), $\mathcal {W}_{\mathrm{alternating}}$ (**b**), and $\mathcal {W}_{\mathrm{all-to-all}}$ (**c**). For each case, we show the results for different optimizers: SGD, Adam, and QNG. Here we fixed $L = 2$. (**d**–**f**) Plot of the fidelity similar as above for different *L* as shown in the colored parentheses (blue star, yellow dagger, green double dagger). Here, blue star is the optimal number of layers for the SGD, yellow dagger is the optimal number of layers for the Adam, and green double dagger is the optimal number of layers for the QNG. We choose the appropriate (blue star, yellow dagger, green double dagger) for each *N* and each $\mathcal {W}$ structure so that the fidelity gets its (possible) highest accuracy.

### Numerical results

#### Single-qubit QST

We first consider reconstructing an abstract single-qubit state encodes in a quantum circuit as shown in the inset Fig. 2. We randomly generate an unknown quantum state $|\psi \rangle = \varvec{U}_3|0\rangle$, where

6 $$\begin{aligned} \varvec{U}_{3}(\theta , \phi , \lambda )= \begin{pmatrix} \cos \frac{\theta }{2} &{}-e^{i \lambda } \sin \frac{\theta }{2} \\ e^{i \phi } \sin \frac{\theta }{2} &{}e^{i(\phi +\theta )} \cos \frac{\theta }{2} \end{pmatrix}, \end{aligned}$$

where we set random with Haar measure $\sin (\theta )/2$, $\phi$, and $\lambda$. To reconstruct $|\psi \rangle$, we set the unitary $\varvec{V}^\dagger (\varvec{\theta }) = R_z(\theta _3)R_x(\theta _2)R_z(\theta _1)$. Indeed, a single-qubit rotation is $R_j(\theta ) = \exp (-i\frac{\theta }{2}\varvec{\sigma }_j), \ j\in \{x, y, z\},$ and $\varvec{\sigma }_j$ is a Pauli matrix applied on the qubit. We train the scheme with 100 iterations using various optimizers and show the cost function versus iteration in the main (Fig. 2). Here, the QNG optimizer gives the best optimization. In the inset figure, we show the trajectory in the Bloch sphere of the reconstructed state $|\phi (\varvec{\theta })\rangle$ under the updated of $\varvec{\theta }$ for two cases of SGD and QNG optimizers. The former needs around 60 iterations for the reconstructed state to reach the true state, while the latter only requires around 6 iterations to reach the same accuracy.

#### Haar random state QST

Now, we focus on a general random Haar state i.e., $|\psi \rangle = \varvec{U}_\mathrm{Haar}|\varvec{0}\rangle$, as shown in Fig. 1b. To reconstruct the state, we use several ansatzes for the entangled gate $\mathcal {W}$ in $\varvec{V}^\dagger (\varvec{\theta })$, including the $\mathcal {W}_\mathrm{chain}, \mathcal {W}_\mathrm{alternating}$, and $\mathcal {W}_\mathrm{all-to-all}$ structures. Refer Fig. 1c for the detailes of these structures, where we used the parameter-dependent controlled *y*-rotation gates to construct them. The circuit’s depth for these structures are $(N+3)L, 4L,$ and $(N+2)L$, respectively. The trainable parameters are $M = 4NL, \lfloor NL/2 \rfloor + 3NL$, and $N(N+5)L/2$, respectively, which are grown linearly with *N*. This is suitable for NISQ devices even for the large number of qubits.

The results are shown in Fig. 3. Let us consider the fidelity between the true Haar state and the reconstructed state as

7 $$\begin{aligned} F(\psi , \phi (\varvec{\theta })) = \big |\langle \phi (\varvec{\theta })|\psi \rangle \big |^2, \end{aligned}$$

which is the overlap between these two states. In Fig. 3a–c, we show the fidelities for different structures of $\mathcal {W}$. For each case, we fix $L = 2$ and examine the three optimizers SGD (blue circle), Adam (yellow square), and QNG (green triangle). We first observe that the SGD optimizer is not good for all $\mathcal {W}$ structures and needs to choose an appropriate learning rate. The fidelities reduce with the increasing *N* and nearly vanish at $N = 6$. In contrast, the Adam optimizer exhibit high fidelities up to $N = 4$ for $\mathcal {W}_\mathrm{chain}$ (a), $N = 5$ for $\mathcal {W}_\mathrm{all-to-all}$ (c), and gradually reduces from $N = 2$ for $\mathcal {W}_\mathrm{alternating}$ (b). Even though it is not stable near the optimal point, the Adam is remarkable for achieving high accuracy in the QST. Furthermore, the QNG optimizer also allows for getting such high accuracy up to $N = 4$ for $\mathcal {W}_\mathrm{chain}$ (a) and even better than the Adam for $\mathcal {W}_\mathrm{alternating}$ (b), while it gradually reduces for $\mathcal {W}_\mathrm{all-to-all}$ (c). This observation can be explained by these own structures: the $\mathcal {W}_\mathrm{all-to-all}$ contains the most number of parameters via the controlled *y*-rotation gates compared to the others, which results in the low accuracy. It is apparent that the QNG optimizer is sensitive to the controlled *y*-rotation gates, where the more controlled *y*-rotation gates, the less efficient QNG optimizer.

Next, to achieve high accuracy for any qubit numbers *N*, we increase the number of layers *L*, while paying attention to the barren plateau^57–61^, i.e., the accuracy of the training process reduces when increasing the parameters space. Figure 3d–f plot the fidelities versus *N*, where for each *N*, the corresponding *L* is shown in the colored parenthesis (blue star, yellow dagger, green double dagger), for the SGD, Adam, and QNG, respectively. The number of layers shown in the parenthesis is the smallest (optimal) *L* required for achieving such high accuracy before it goes down due to the barren plateau. As can be seen from the figure, the Adam method allows for reaching the maximum fidelity (results are shown up to $N = 5$ for all $\mathcal {W}$ structures) with a suitable *L* as shown in the middle position of the parenthesis. Similarly, we can reach high accuracy with the QNG optimizer up to $N = 4$ when choosing an appropriate *L* as shown in the last position of the parenthesis. For the SGD, it is intractable for achieving high accuracy, such as for $\mathcal {W}_\mathrm{alternating}$. Even though the relation between *N* and the required *L* is not clear, interestingly, we can see from the results up to $N = 5$, the required *L* is also around 5 (more *L* is redundancy or may reduce the accuracy due to the barren plateau, see details in “Methods” section).

We only simulate up to $N = 5$. However, for larger *N*, the scheme still works well. Evidently, in Fig. 3d–f, we enhance high fidelity with an appropriate optimizer for every *N* up to 5. Following the procedure in “Methods” section, we can entirely expand to a larger *N* while still maintaining high fidelity.

#### Compare to the shadow tomography protocol

Finally, we address the merit of our UC-QST approach and the shadow tomography protocol^48,49^, a recent promising method in this regime. A shadow tomography protocol is given as follows^49^: (1) initially prepare a random unknown quantum state $\rho$, and the task ahead is to predict a target function underlying the state from its shadow, (2) randomly pick up a unitary $\varvec{U}_k$ in a *T*-tuple $\mathcal {U}$, i.e., $\mathcal {U} = \{\varvec{U}_1, \varvec{U}_2, \ldots , \varvec{U}_T\}$ then apply it to the initial state to transform $\rho \mapsto \varvec{U} \rho \varvec{U}^\dagger$, (3) measure the evolved state in the computational basis $|b\rangle = \{|0\rangle , |1\rangle \}^N$. Steps (2) and (3) are repeated for a certain number of measurements. For each measurement, we get a random classical snapshot

8 $$\begin{aligned} \sigma _{k,b} = \varvec{U}^\dagger _k |b\rangle \langle b|\varvec{U}_k. \end{aligned}$$

We then define an invertible channel matrix

9 $$\begin{aligned} \mathcal {M}(\rho ) =\mathbb {E}_k \sum _b \mathrm{Tr}(\sigma _{k,b}\ \rho ) \cdot \sigma _{k,b}, \end{aligned}$$

where $\mathbb {E}_k$ is the average over $\varvec{U}_k$, with a corresponding pick-up probability. Let $\mathcal {M}^{-1}$ exists, and let $p_k$ is the probability of picking up a unitary $\varvec{U}_k$, then we can reconstruct a (non-normalized) state as

10 $$\begin{aligned} \check{\rho }= \sum _{k}p_k \sum _b \mathrm{Tr}(\sigma _{k,b}\ \rho )\cdot \mathcal {M}^{-1} (\sigma _{k,b}), \end{aligned}$$

which is the classical shadow of the original unknown state $\rho$. For the transformation $\varvec{U}$ belongs to a family of the global Clifford gates, i.e., $\varvec{U}\in \mathcal {U}_{C} = \{ \mathrm{CNOT, Hadamard, S\_gate, T\_gate}\}$, refer to Random Clifford measurements, the reconstructed state explicitly yields^49^

11 $$\begin{aligned} \check{\rho }= (2^N+1) \varvec{U}^\dagger |b\rangle \langle b|\varvec{U} -\varvec{I}. \end{aligned}$$

For the transformation $\varvec{U}$ belongs to the random Pauli gates, such as $\varvec{U} \in \mathcal {U}_P = \{\varvec{\sigma }_x, \varvec{\sigma }_y, \varvec{\sigma }_z, \ldots \}$, refer to Random Pauli measurements, it straightforwardly yields^49^

12 $$\begin{aligned} \check{\rho }= \bigotimes _{j = 1}^N \Bigl ( 3\varvec{U}_j^\dagger |b_j\rangle \langle b_j|\varvec{U}_j -\varvec{I} \Bigr ), \end{aligned}$$

for $$b = (b_1, \ldots , b_N) \in \{0,1\}^N$$.

For comparing the shadow tomography with the UC-QST scheme, we apply the Random Pauli measurements and consider the prediction of a linear function as a figure of merit for the accuracy. A global observable $\mathcal {\varvec{Z}} \equiv \varvec{\sigma }_z^{\otimes N}$, gives the predicted (linear) expectation value as

13 $$\begin{aligned} {\check{z}} = \mathrm{Tr} (\mathcal {\varvec{Z}}\check{\rho }), \text { that obeys } \mathbb {E}[\check{z}] = \mathrm{Tr}(\mathcal {\varvec{Z}}\rho ). \end{aligned}$$

The fluctuation (distribution around the true expectation value) of the predicted expectation value is given by the variance Var$[\check{z}]$ as

14 $$\begin{aligned} \mathrm{Var}[\check{z}] = \mathbb {E} \bigl [\bigl (\check{z} - \mathbb {E}[\check{z}] \bigl )^2\bigr ] = \bigl [\bigl ( \mathrm{Tr}(\mathcal {\varvec{Z}}\check{\rho }) - \mathrm{Tr}(\mathcal {\varvec{Z}}\rho ) \bigl )^2\bigr ]. \end{aligned}$$

**Figure 4:**
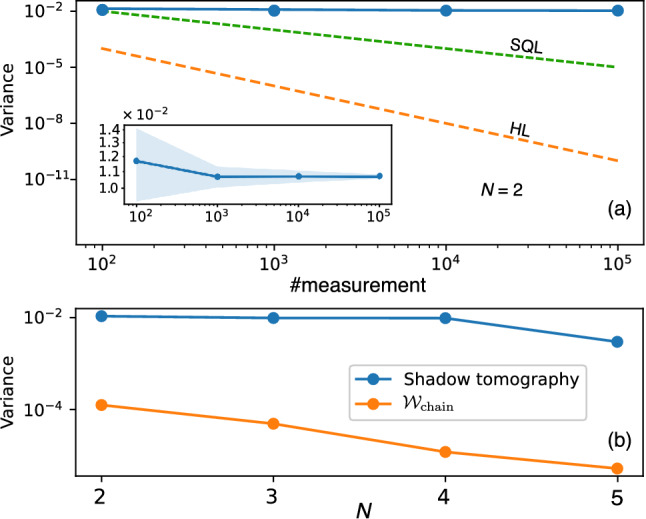
Comparison between the UC-QST approach and the shadow tomography method. (**a**) Log–log plot of the variance Var$[\check{z}]$ versus the number of repeated measurements (#measurement) using the shadow tomography. The standard quantum limit (SQL) and Heisenberg limit (HL) are shown for comparison purposes. The number of qubits is fixed at $N = 2$. Inset: Zoom-in the variance, where the blue area is the standard deviation after talking ten runs. (**b**) Plot of variance for the shadow tomography (blue) and UC-QST (orange). For the shadow tomography, we fix #measurement $= 10^5$, and for the UC-QST, we use the structure of $\mathcal {W}_\mathrm{chain}$ and the Adam optimizer, the number of shots is $10^4$.

In Fig. 4a, we show the variance Var$[\check{z}]$ as a function of the number of measurements for the shadow tomography. The variance slightly decreases when increasing the number of measurements from $10^2$ to $10^5$. See the inset figure for the detailed zoom-in. The result is compared with the standard quantum limit (SQL), i.e., SQL = 1/ #measurement, and the Heisenberg limit (HL), i.e., HL = 1/ (#measurement)$^2$. Here, the variance does not beat the SQL nor HL.

In Fig. 4b, we compare the variances obtained from the shadow tomography and the UC-QST for the different number of qubits *N*. For the shadow tomography, we fix #measurement $= 10^5$. For the UC-QST, we consider the $\mathcal {W}_\mathrm{chain}$ structure with the Adam optimizer as an example. The number of shots is fixed at $10^4$. It can be seen that the UC-QST offers a better result over 100 times than the resulting shadow tomography.

## Discussion

We discuss some features of the UC-QST and shadow tomography in the previous section. The shadow tomography only allows predicting target functions, such as expectation values, entanglement entropies, correlation functions, and so on^49^, while maintaining the precision. Whereas the UC-QST allows for reconstructing the entire quantum state up to a phase shift. Both schemes allow for predicting properties of quantum states or quantum states with fewer measurements compared to standard quantum tomography. Another remarkable feature is that the efficiency of the shadow tomography protocol depends on the random choice of the unitaries in an ensemble $\mathcal {U}$, while the efficiency of the UC-QST scheme relies on the choice of different ansatzes and optimizes. Finally, we emphasize that the comparison in this section only provides a very first glance about the two approaches. We need to further characterize these features in future works for more concrete evaluation.

Furthermore, the idea of UC-QST resembles the self-guided quantum tomography (SGQT)^62^ and single-shot measurement (SSM)^46,47^. These previous approaches also iteratively search the estimated state to converge to the true state. However, the trainable unitary topologies and optimization algorithms are different. The SGQT uses a simultaneous perturbation stochastic approximation^63^ to optimize the cost function, while the SSM trains a learning unitary to be a fiducial (known) state that converges to the true state. Here, we apply the universal compilation technique to train a learning unitary to be the target unitary.

Finally, we emphasize that the current method is suitable only for pure-state tomography and not for general mixed-state tomography.

## Methods

### Training process

The training process is a hybrid protocol as illustrated in Fig. 1a: a set of unitary gates $\varvec{U}$ followed by $\varvec{V}^\dagger$ are applied onto the circuit and the final state is measured afterwards. The results are sent to the classical counterpart to compute the corresponding cost function and then update new parameters $\varvec{\theta }$ using a suitable optimizer protocol until it reaches convergence.

We use gradient-based optimizations to iteratively update the parameters $\varvec{\theta }$ and minimize the cost function. To do that, we need to calculate the derivative $\partial \mathcal {C}(\varvec{\theta })/ \partial \theta _{j}$ w.r.t $\theta _j$ in the *j*th gate for every $\theta _j\in \varvec{\theta }$. We compute two cases as follows. First, if the *j*th gate is a single-qubit rotation gate, i.e., $\exp (-i\theta _j\varvec{\sigma }_k/2), k \in \{x,y,z\}$, then using the standard (two-term) parameter-shift rule^64,65^, we have

15 $$\begin{aligned} \dfrac{\partial \mathcal {C}(\varvec{\theta })}{\partial \theta _j}&= -\dfrac{1}{2\mathcal {C}(\varvec{\theta })} \dfrac{\partial p_{0}(\varvec{\theta })}{\partial \theta _j}\nonumber \\&=-\frac{1}{2\mathcal {C}(\varvec{\theta })} \frac{1}{2\sin (s)} \Big [p_0(\varvec{\theta }+ s\varvec{e}_j) - p_0(\varvec{\theta }- s\varvec{e}_j)\Big ], \end{aligned}$$

where *s* denotes an arbitrary shift, and $\varvec{e}_j$ is the *j*th unit vector, or in other words, we only add *s* to $\theta _j$. Second, if the *j*th gate is a controlled rotation gate, i.e., $CR_y(\theta _j)$, then using the four-term parameter-shift rule^66^, we partially compute

16 $$\begin{aligned} \dfrac{\partial p_{0}(\varvec{\theta })}{\partial \theta _j}&= d_+ \Big [p_0(\varvec{\theta }+ a\varvec{e}_j) - p_0(\varvec{\theta }- a\varvec{e}_j)\Big ] \nonumber \\&\quad - d_- \Big [p_0(\varvec{\theta }+ b\varvec{e}_j) - p_0(\varvec{\theta }- b\varvec{e}_j)\Big ], \end{aligned}$$

where $d_\pm = (\sqrt{2}\pm 1)/4\sqrt{2};\ a= \pi /2;\ b = 3\pi /2$. Then, we get $\frac{\partial \mathcal {C}(\varvec{\theta })}{ \partial \theta _j} = -\frac{1}{2\mathcal {C}(\varvec{\theta })} \frac{\partial p_{0}(\varvec{\theta })}{\partial \theta _j}$.

To compute new parameters, we use several optimizers in all experiments: Standard gradient descent (SGD), Adam gradient descent^55^, and Quantum natural gradient (QNG)^56^.

The formula for SGD reads

17 $$\begin{aligned} \varvec{\theta }^{t+1} =\varvec{\theta }^{t}-\alpha \nabla _{\varvec{\theta }}\mathcal {C}(\varvec{\theta }), \end{aligned}$$

where $\nabla _{\varvec{\theta }} \mathcal {C}(\varvec{\theta }) = \big ( \partial _{\theta _1}\mathcal {C}(\varvec{\theta }), \partial _{\theta _2}\mathcal {C}(\varvec{\theta }), \cdots , \partial _{\theta _M}\mathcal {C}(\varvec{\theta }) \big )^\mathrm{T}$ for *M* training parameters, and $\alpha$ is the learning rate. In comparison, Adam is a non-local averaging optimizer that allows adapting the learning rate but requires more steps than the SGD

18 $$\begin{aligned}&\varvec{\theta }^{t+1}=\varvec{\theta }^{t} -\alpha \frac{\hat{m}_{t}}{\sqrt{\hat{v}_{t}} + \epsilon }, \end{aligned}$$

where $m_{t}=\beta _{1} m_{t-1} +\left( 1-\beta _{1}\right) \nabla _{\varvec{\theta }}\mathcal {C}(\varvec{\theta }), v_{t}=\beta _{2} v_{t-1}+(1-\beta _{2}) \nabla _{\varvec{\theta }}^2\mathcal {C}(\varvec{\theta }), \hat{m}_{t}=m_{t} /\left( 1-\beta _{1}^{t}\right) , \hat{v}_{t}=v_{t} /\left( 1-\beta _{2}^{t}\right) ,$ with the hyper-parameters are chosen as $\alpha = 0.2, \beta _1 = 0.8, \beta _2 = 0.999$ and $\epsilon = 10^{-8}$. Finally, the QNG is defined by

19 $$\begin{aligned} \varvec{\theta }^{t+1}=\varvec{\theta }^{t}-\alpha g^+\nabla _{\varvec{\theta }}\mathcal {C}(\varvec{\theta }), \end{aligned}$$

where $g^+$ is the pseudo-inverse of a Fubini-Study metric tensor *g*^67^. Assume that we can group $\varvec{\theta }$ into $\mathcal {L}$ layers, i.e., $\varvec{\theta }= \varvec{\theta }^{(1)}\oplus \varvec{\theta }^{(2)}\oplus \cdots \oplus \varvec{\theta }^{(\mathcal {L})}$, so that in each layer $\varvec{\theta }^{(\ell )} = \{\theta ^{(\ell )}_1, \theta ^{(\ell )}_2,\ldots ,\theta ^{(\ell )}_{M^{(\ell )}} \big |\ \sum _\ell M^{(\ell )} = M\}$, any two of unitaries satisfy $[\varvec{G}_i^{(\ell )}, \varvec{G}_j^{(\ell )}]=\delta _{ij}$. Then, the metric tensor *g* gives^68^

20 $$\begin{aligned} g = \left( \begin{array}{cccc} \left[ \begin{array}{c} g^{(1)} \end{array}\right] &{} &{} &{} \varvec{0}\\ &{} \left[ \begin{array}{c} g^{(2)} \end{array}\right] &{} \\ &{} &{} \ddots \\ \varvec{0} &{} &{} &{} \left[ \begin{array}{c} g^{(\mathcal {L})} \end{array}\right] \end{array}\right) \end{aligned}$$

where an element $g_{ij}^{(\ell )}$ of $g^{(\ell )}$ reads

21 $$\begin{aligned} g_{i j}^{(\ell )} = \mathrm{Re}\big [ \langle \partial _i\psi _{\ell } | \partial _j \psi _{\ell }\rangle - \langle \partial _i\psi _{\ell }| \psi _{\ell } \rangle \langle \psi _{\ell }|\partial _j \psi _{\ell }\rangle \big ], \end{aligned}$$

where $|\psi _{\ell }\rangle$ is the quantum state at the $\ell$th layer. For unitary $\varvec{G}_i^{(\ell )} = e^{-i\theta _i^{(\ell )} \varvec{K}_i^{(\ell )}}$, e.g., a rotation gate, such that $[\varvec{G}_i^{(\ell )}, \varvec{K}_i^{(\ell )}] = 0$, then $g_{i j}^{(\ell )}$ is recast as^68^

22 $$\begin{aligned} g_{i j}^{(\ell )}&= \mathrm{Re}\big [\langle \psi _{\ell -1} |\varvec{K}_{i} \varvec{K}_{j}| \psi _{\ell -1}\rangle \nonumber \\&\quad - \langle \psi _{\ell -1}|\varvec{K}_{i}| \psi _{\ell -1} \rangle \langle \psi _{\ell -1}|\varvec{K}_{j}| \psi _{\ell -1}\rangle \big ]. \end{aligned}$$

See a detailed example of computing a tensor metric *g* below.

Each optimizer has its own pros and cons: (1) the SGD is simple but low coverage, one must choose a proper learning rate to achieve the best result, (2) the Adam allows to automatically adapt the learning rate and fast coverage but it is noisy near the optimal point, and (3) the QNG is better than other optimizers but also requires more computational cost regards to quantum circuits. While the SGD and Adam do not depend on quantum states and work for any classical data types, including the probabilities, the QNG optimizes the parameters towards the geometry of evolved quantum states and is thus expected to offer better and faster optimization. We conduct these optimizers based on their advantages and disadvantages and compare the results. They also serve as a test bed and reference for future works.

This work implements the numerical experiments using various configurations described above to train the variational models and compare them together. The numerical results are executed by Qiskit open-source package, version 0.24.0, which is available to run on all platforms. For each experiment, to get the probability $p_0$ we execute $10^4$ shots using the *qasm* simulator backend. The number of iterations for every training process is fixed at 400, except for others shown in the text. It is sufficient for the cost function to converge for all data shown in the text. The experiments are then scaled up to 6 qubits for quantum state tomography to demonstrate the scalability. Furthermore, after the training process, we can reproduce the unknown state by applying $\varvec{V}(\varvec{\theta }^*)$ into the initial state $|\varvec{0}\rangle$, and use it for further applications and other statistical computations.

### Complexity

In terms of complexity, to execute the parameter-shift rule in Eq. (15), the quantum circuit executes $2M + 1$ times, one *M* times to compute $p_0(\varvec{\theta }+ s\varvec{e}_i)$, one *M* times to compute $p_0(\varvec{\theta }- s\varvec{e}_i)$, and one time to compute $p_0(\varvec{\theta })$. Furthermore, a single evaluation requires executing the circuit for a constant number of shots to reach a certain precision, and each execution involves around *G* gate operations. So, the complexity of each iteration is $\mathcal {O}[(2M+1)G]$. Similarly, the complexity for an iteration with four-term parameter-shift rule is $\mathcal {O}[(4M+1)G]$.

Ideally, after each step, the cost function will decrease with a linear or logarithmic speed regarding the number of iterations. However, the variational circuit always offers a lower bound of the cost function during the training process. In particular, this bound increases by the number of qubits *N*, which means the problem will be harder according to the size of the system

23 $$\begin{aligned} \mathcal {C}(\varvec{\theta }) \ge \textit{poly}(N). \end{aligned}$$

The complexity of the ansatz $\varvec{V}(\varvec{\theta })$ is another challenge. Its current structure is fixed into the chain, alternating, and all-to-all. However, the structure also needs to optimize in future works, e.g., using Genetic Algorithms for generating a compressed ansatz $\varvec{V}(\varvec{\theta })$ that can work well on the current NISQ devices for the large number of qubits.

### Fubini-Study tensor metric

**Figure 5:**
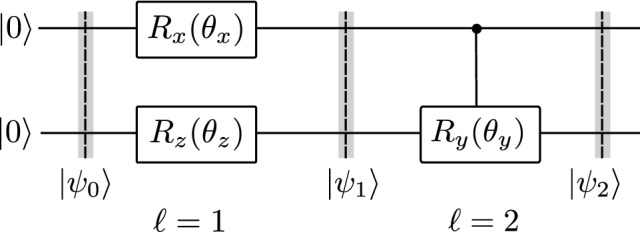
An example quantum circuit for evaluating the Fubini-Study tensor metric. The circuit starts with the initial state $|\psi _0\rangle = |00\rangle$ and evolves under a set of gates $R_x(\theta _x), R_z(\theta _z)$ in layer 1, and $CR_y(\theta _y)$ in layer 2. Detailed of the Fubini-Study tensor metric evaluation for this circuit is provided in the text.

We provide a practical example of how to compute a Fubini-Study tensor metric. Let us consider a concrete circuit as shown in Fig. 5. It consists of $R_x = \exp (-i\frac{\theta _x}{2}\varvec{\sigma }_x)$, $R_z = \exp (-i\frac{\theta _z}{2}\varvec{\sigma }_z)$, and $CR_y = |0\rangle \langle 0|\otimes \varvec{I}_2 + |1\rangle \langle 1|\otimes \exp (-i\frac{\theta _y}{2}\varvec{\sigma }_y)$. Since $[R_x, R_z] = 0$ (because they act on different qubits), we can group them into one layer (layer 1), with $\varvec{\theta }^{(1)} = \{\theta ^{(1)}_0, \theta ^{(1)}_1\} = \{\theta _x, \theta _z\}$, and put $CR_y$ into another layer (layer 2), with $\varvec{\theta }^{(2)} = \{\theta ^{(2)}_0\} = \{\theta _y\}$. The tensor metric *g* explicitly yields

24 $$\begin{aligned} g = \begin{pmatrix} g_{xx}^{(1)} &{} g_{xz}^{(1)} &{} 0\\ g_{zx}^{(1)} &{} g_{zz}^{(1)} &{} 0\\ 0 &{} 0 &{} g_{yy}^{(2)} \end{pmatrix}. \end{aligned}$$

The quantum states are explicitly expressed as

25 $$\begin{aligned} |\psi _0\rangle&= |00\rangle ,\; \end{aligned}$$

26 $$\begin{aligned} |\psi _1\rangle&= e^{-i\frac{\theta _x}{2}\varvec{\sigma }_x\otimes \varvec{I}_2} e^{-i\frac{\theta _z}{2}\varvec{I}_2\otimes \varvec{\sigma }_z} |\psi _0\rangle , \end{aligned}$$

27 $$\begin{aligned} |\psi _2\rangle&= \big [|0\rangle \langle 0|\otimes \varvec{I}_2 + |1\rangle \langle 1|\otimes e^{-i\frac{\theta _y}{2}\varvec{\sigma }_y}\big ] |\psi _1\rangle . \end{aligned}$$

The elements $g^{(1)}_{ij}$ is given through Eq. (22) as

$$\begin{aligned} g^{(1)}_{xx}&= \langle \psi _0|\varvec{K}_x^2|\psi _0\rangle -\langle \psi _0|\varvec{K}_x|\psi _0\rangle ^2 = \dfrac{1}{4}\;,\\ g^{(1)}_{xz}&= \langle \psi _0|\varvec{K}_x\varvec{K}_z|\psi _0\rangle -\langle \psi _0|\varvec{K}_x|\psi _0\rangle \langle \psi _0|\varvec{K}_z|\psi _0\rangle = 0\;,\\ g^{(1)}_{zx}&= \langle \psi _0|\varvec{K}_z\varvec{K}_x|\psi _0\rangle -\langle \psi _0|\varvec{K}_z|\psi _0\rangle \langle \psi _0|\varvec{K}_x|\psi _0\rangle = 0\;,\\ g^{(1)}_{zz}&= \langle \psi _0|\varvec{K}_z^2|\psi _0\rangle -\langle \psi _0|\varvec{K}_z|\psi _0\rangle ^2 = 0\;, \end{aligned}$$

where $$\varvec{K}_x = \frac{\varvec{\sigma }_x\otimes \varvec{I}_2}{2}$$ and $$\varvec{K}_z = \frac{\varvec{I}_2\otimes \varvec{\sigma }_z}{2}$$.

Next, we calculate $g^{(2)}_{yy}$. Starting from Eq. (21) in the main text, we derive

28 $$\begin{aligned} |\partial _{\theta _y}\psi _2\rangle = -i|1\rangle \left\langle 1| \otimes \dfrac{\varvec{\sigma }_y}{2}e^{-i\frac{\theta _y}{2}\varvec{\sigma }_y} |\psi _1\right\rangle . \end{aligned}$$

Then, we get

29 $$\begin{aligned} g^{(2)}_{yy}&= \langle \psi _1|\varvec{K}_y^2|\psi _1\rangle -\langle \psi _1|\varvec{K}_y|\psi _1\rangle ^2\nonumber \\&= \dfrac{1}{4}\sin ^2\big (\textstyle \frac{\theta _x}{2}\big ), \end{aligned}$$

where $\varvec{K}_y = |1\rangle \langle 1|\otimes \frac{\varvec{\sigma }_y}{2}$. To derive expectation values in Eq. (29), we prepare $|\psi _1\rangle$ as in Fig. 5, then measure $\langle \psi _1|\varvec{K}_y^2|\psi _1\rangle = \frac{1}{4}\langle \psi _1|\big (|1\rangle \langle 1 |\otimes \varvec{I}_2\big )|\psi _1\rangle$ and $\langle \psi _1|\varvec{K}_y|\psi _1\rangle = \frac{1}{2}\langle \psi _1|\big (|1\rangle \langle 1 |\otimes \varvec{\sigma }_y\big )|\psi _1\rangle$. Finally, we obtain the tensor metric *g*

30 $$\begin{aligned} g = \begin{pmatrix} \frac{1}{4} &{} 0&{} 0\\ 0 &{} 0 &{} 0\\ 0 &{} 0 &{} \frac{1}{4}\sin ^2(\frac{\theta _x}{2}) \end{pmatrix}. \end{aligned}$$

### Supported data for QST

**Figure 6:**
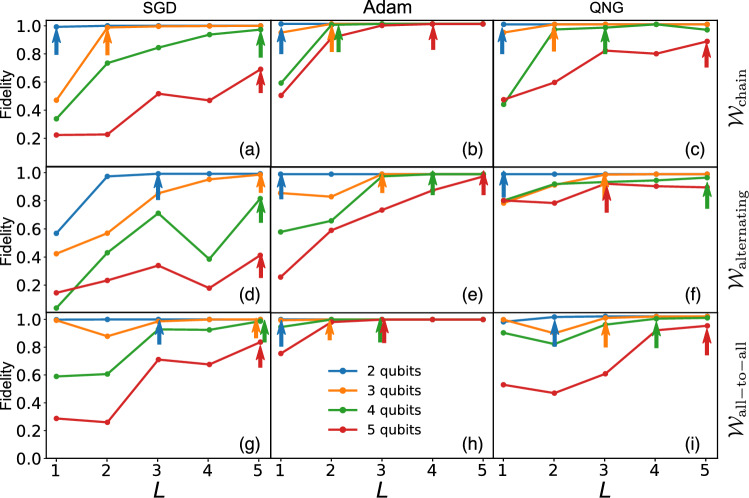
Plot of fidelity as a function of *L* for different $\mathcal {W}$ structures and different optimizers. (**a**) ($\mathcal {W}_\mathrm{chain}$, SGD), (**b**) ($\mathcal {W}_\mathrm{chain}$, Adam), (**c**) ($\mathcal {W}_\mathrm{chain}$, QNG), (**d**) ($\mathcal {W}_\mathrm{alternating}$, SGD), (**e**) ($\mathcal {W}_\mathrm{alternating}$, Adam), (**f**) ($\mathcal {W}_\mathrm{all\_ to\_ all}$, QNG), (**g**) ($\mathcal {W}_\mathrm{all\_ to\_ all}$, SGD), (**h**) ($\mathcal {W}_\mathrm{all\_ to\_ all}$, Adam), (**i**) ($\mathcal {W}_\mathrm{all\_ to\_ all}$, QNG).

We discuss more data supporting the results in Fig. 3d–f in the main text. As we discussed above, the accuracy can be improved when increasing the number of layers *L*. However, we cannot increase *L* arbitrarily large and need to stop at an optimal point. We define the optimal *L* as the smallest number of layers that, at the next layer, the accuracy saturates or starts to reduce. In Fig. 6 below, we discuss the optimal *L* for various cases, where we mark the optimal *L* with colored arrows. See also Table 1 below.

**Table 1:** Number of optimal layers *L* taking from Fig. 6.

Structure	Optimizer	*N*			
		2 ()	3 ()	4 ()	5 ()
$\mathcal {W}_\mathrm{chain}$	SGD	$L = 1$	$L = 2$	$L = 5$	$L = 5$
	Adam	$L = 1$	$L = 2$	$L = 2$	$L = 4$
	QNG	$L = 1$	$L = 2$	$L = 3$	$L = 5$
	in Fig 3d	()	()	()	()
$\mathcal {W}_\mathrm{alternating}$	SGD	$L = 3$	$L = 5$	$L = 5$	$L = 5$
	Adam	$L = 1$	$L = 3$	$L = 4$	$L = 5$
	QNG	$L = 1$	$L = 3$	$L = 5$	$L = 3$
	In Fig. 3e	()	()	()	()
$\mathcal {W}_\mathrm{all-to-all}$	SGD	$L = 3$	$L = 5$	$L = 5$	$L = 5$
	Adam	$L = 1$	$L = 2$	$L = 3$	$L = 3$
	QNG	$L = 2$	$L = 3$	$L = 4$	$L = 5$
	In Fig. 3f	()	()	()	()

From the results here, we trace out the optimal *L* as shown in Fig. 3 in the main text.

## Data Availability

Data are available from the corresponding authors upon reasonable request.

## Code availability

All codes used to produce the findings of this study are available at: https://github.com/vutuanhai237/UC-VQA.
